# Comparing fetal phantoms with surrogate organs in female phantoms during CT exposure of pregnant patients

**DOI:** 10.1007/s13246-024-01383-3

**Published:** 2024-01-11

**Authors:** Mohamed Khaldoun Badawy, Kashish Kashish, Shay Payne, Maeve Masterson

**Affiliations:** 1 Monash Health Imaging, Monash Health, 246 Clayton Road, Clayton, VIC 3168 Australia; 2 Department of Medical Imaging and Radiation Sciences, School of Primary and Allied Health Care, Faculty of Medicine, Nursing and Health Sciences, Monash University, Clayton, VIC 3800 Australia; 3 School of Life and Environmental Sciences, Deakin University, Burwood, VIC 3125 Australia

**Keywords:** Computed tomography, Pregnancy, Dosimetry, Phantoms, Radiation safety

## Abstract

With the rising use of Computed Tomography (CT) in diagnostic radiology, there are concerns regarding radiation exposure to sensitive groups, including pregnant patients. Accurately determining the radiation dose to the fetus during CT scans is essential to balance diagnostic efficacy with patient safety. This study assessed the accuracy of using the female uterus as a surrogate for fetal radiation dose during CT imaging. The study used common CT protocols to encompass various scenarios, including primary beam, scatter, and partial exposure. The computational program NCICT was used to calculate radiation doses for an adult female and a fetus phantom. The study highlighted that using the uterus for dose estimation can result in consistent underestimations of the effective dose, particularly when the fetus lies within the primary radiation beam. These discrepancies may influence clinical decisions, affecting care strategies and perceptions of associated risks. In conclusion, while the female uterus can indicate fetal radiation dose if the fetus is outside the primary beam, it is unreliable when the fetus is within the primary beam. More reliable abdomen/pelvic organs were recommended.

## Introduction

Computed tomography is widely used in diagnostic radiology, producing three-dimensional images with superior soft tissue contrast and speed compared to planar radiographic methods [1]. Although advancements in CT technology have significantly lowered radiation doses, patients undergoing a chest CT may still receive radiation levels up to 100 times greater than those from standard chest X-rays in some instances [2]. The use of CT scans has markedly increased, as demonstrated by a 211% rise from 2001 to 2019 in Australia [3]. This growing accessibility and utilisation of CT imaging pose important considerations, particularly for radiosensitive groups such as pregnant women.

The fetus, especially in early developmental stages, is highly sensitive to the adverse effects of ionising radiation [4, 5]. Exposure to radiation doses exceeding 100mGy during fetal organogenesis can lead to inhibited growth and cognitive risks, with the most significant impact occurring in the first trimester. Additional risks include miscarriage, developmental anomalies, and potential carcinogenic or mutagenic effects. While it is uncommon for diagnostic imaging to reach these dose levels, the possibility exists, particularly in extreme cases or serial imaging. Therefore, minimising exposure to unjustified or repeated CT exams in pregnant patients is crucial, especially when estimates of fetal dose are high. Given these risks, determining the radiation dose and its associated risks to the fetus becomes essential in clinical practice. Accurate quantification may influence the decision-making process in choosing between CT and other imaging modalities [6]. Dose estimation plays a vital role in clinical practice, aiding healthcare professionals in justifying the necessity of CT exams and equipping them with essential information to discuss potential fetal risks with patients, particularly when patient consent is required.

Combined with modern risk models, mathematical phantoms offer insights into fetal radiation exposure during pregnancy [7]. These three-dimensional models, often derived from CT or other imaging data, emulate human anatomy. While commercial software has historically relied on basic non-pregnant female phantoms, equating uterus dose to fetal dose [8, 9], this approximation can be flawed due to the fetus’s dynamic position throughout gestation. Angel et al.‘s methodology, which uses tube current and exposure (mAs) as a scaling factor, highlighted that commercial estimates often overstate the fetal dose despite reasonable agreement with Monte Carlo results [10]. Meanwhile, Lopez-Rendon et al. highlighted a significant disparity, between −15.9 and 40%, when comparing commercial software and voxel models of pregnant patients, especially when adjusting for tube current modulation [11]. However, employing Monte Carlo techniques demands significant resources and expertise.

The National Cancer Institute introduced a user-friendly dosimetry system for CT, NCICT [12]. This software uses computational phantoms developed by the International Commission on Radiological Protection (ICRP) which incorporates more anatomically accurate patient models, including pregnant variants across gestational stages. It facilitates dose estimates using lookup tables derived from Monte Carlo simulations, tailored to accommodate different gestational ages and technique factors. This study aims to compare the radiation doses using the uterus and other abdominal/pelvic organs in a female phantom with those determined using an anatomically detailed fetal phantom during CT imaging.

## Methods

In this study, the authors employed protocols from the Facility Reference Level audits, ensuring a representation of the most used protocols on site. These protocols encompass a variety of procedures, exposing the fetus to scenarios such as the primary beam, scatter radiation, and potential partial exposure—as observed in CT chest scans during certain gestational periods. Table 1 lists the protocols and their average CTDI values, derived from averaging the median CTDI across all scanners within the hospital network with scan ranges in line with clinical protocols.

**Table 1:** Average CTDIvol (mGy) values and scan range used for the CT imaging protocols used in this study

Protocol	Scan start	Scan end	CTDI_vol_ FRL (mGy)
CT Abdomen and pelvis	Above the diaphragm	Below the lesser trochanter of the femur	7.8
CT Brain	Vertex of skull	Mid C1 vertebra	33.0
CT Chest	Above the diaphragm	Adrenal glands	6.0
CT Chest, abdomen & pelvis	Top of lung apices	Below the lesser trochanter of the femur	7.0
CT Kidneys, ureters & bladder	Above the kidneys	Below the lesser trochanter of the femur	4.8
CT Lumbosacral spine	Middle of T12 vertebra	S2 vertebra	16.5
CT Cervical spine	Base of skull	T2 vertebra	15.7

**Fig. 1:**
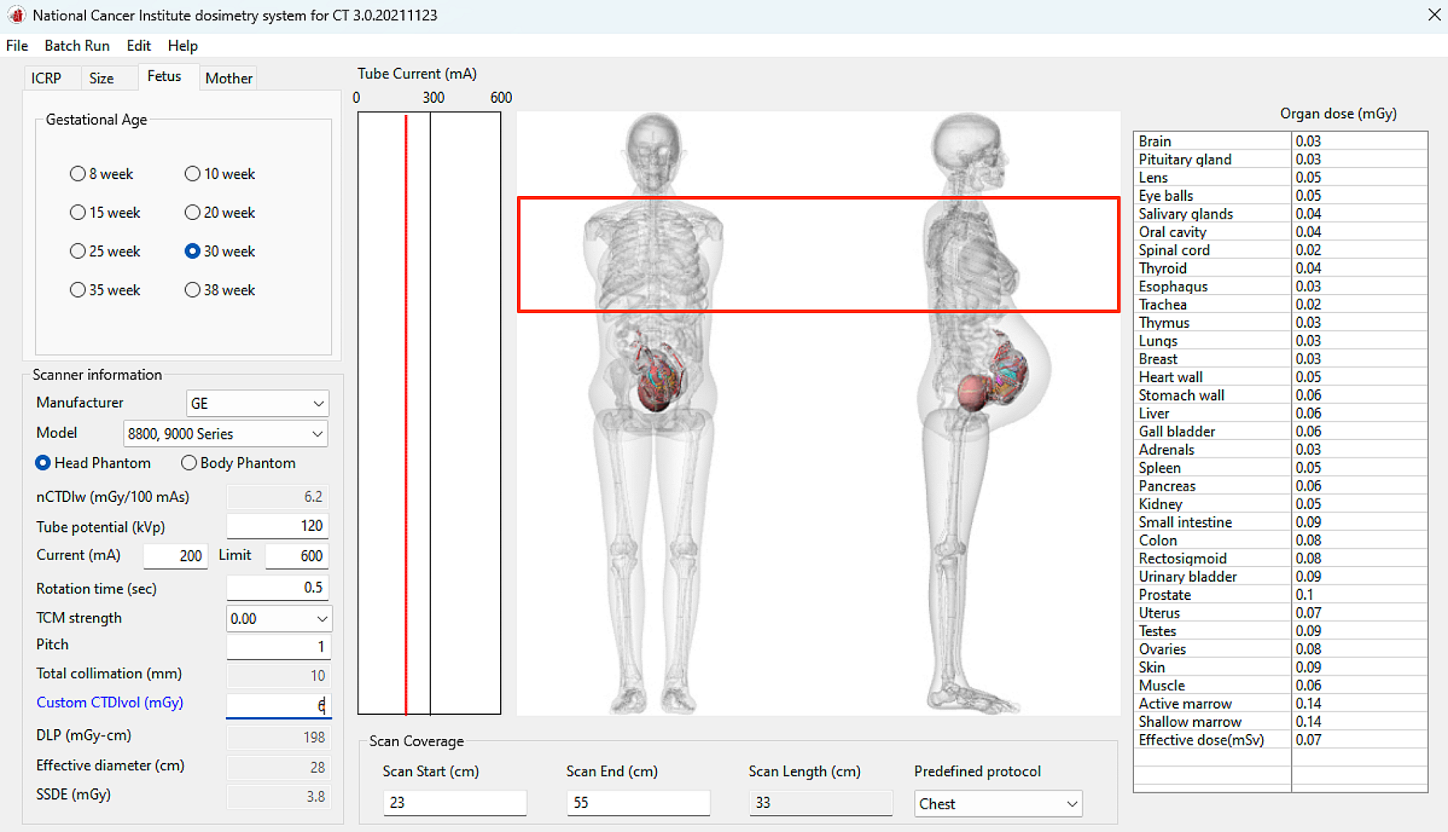
The user interface of the NCICT program utilising the fetal specific phantoms

The radiation dose of the uterus and other organs was calculated using the computational program NCICT [12], user interface shown in Fig. 1. Organ doses for an adult female and fetus phantom were computed using the anatomical phantoms within NCICT by adjusting specific parameters. Parameters ‘Age Group’ and ‘Gender’ were set to an adult female, with ‘Height’ and ‘Weight’ fixed to standard Reference Body Size. The ‘Scanner Information’ remained consistent across calculations, with all parameters fixed except for Custom CTDI_vol_ (mGy). The manufacturer was consistently set as ‘GE’, with ‘Model’ defined as ‘GE AVERAGE’. The ‘Body Phantom’ was standard for all protocols, except the CT Brain protocol, which used the ‘Head Phantom’. Adjustments to ‘scan start’ and ‘scan end’ values were necessary to ensure correct anatomical coverage during gestation, which impacted the overall ‘scan length’. Custom CTDI_vol_ (mGy) values, as listed in Table 1, were input across varying gestational ages (Weeks 8, 10, 15, 20, 25, 30, 35, and 38) for each protocol. The tube voltage was 120 kVp for all protocols. All data were recorded for each protocol and phantom combination, including phantom type, age/gestational age, scan range values, CTDIvol, and organ dose (mGy).

The processing, analysis, and graphical representations were conducted using Python 3.8 as the primary programming language. For data manipulation and analysis, the pandas library (version 1.1.5) was employed. Data visualisation was achieved using Matplotlib (version 3.3.3), complemented by Seaborn (version 0.11.0) for advanced visualisation techniques. Numerical operations were facilitated by NumPy (version 1.19.5). Line plots were generated to visually illustrate the relationship between gestational ages and the corresponding effective doses for the fetus. Concurrently, line plots were created to depict the uterus doses for the adult female phantom. These datasets were merged to facilitate visual comparison between the uterus and fetal effective doses across various protocols. The difference between all abdomen/pelvis organ doses in the female phantom and the effective dose for the fetal phantom was calculated using Eq. (1). This analysis covered every protocol and gestational stage included in this study. Heatmaps were produced for each protocol to visualise the dose relationships.

1 $$ \% \; difference = \frac{Organ \; dose \;\left(mGy\right) - Fetal \; dose \;\left(mSv\right)}{Fetal \; dose \;\left(mSv\right)} \times100$$

## Results

**Fig. 2:**
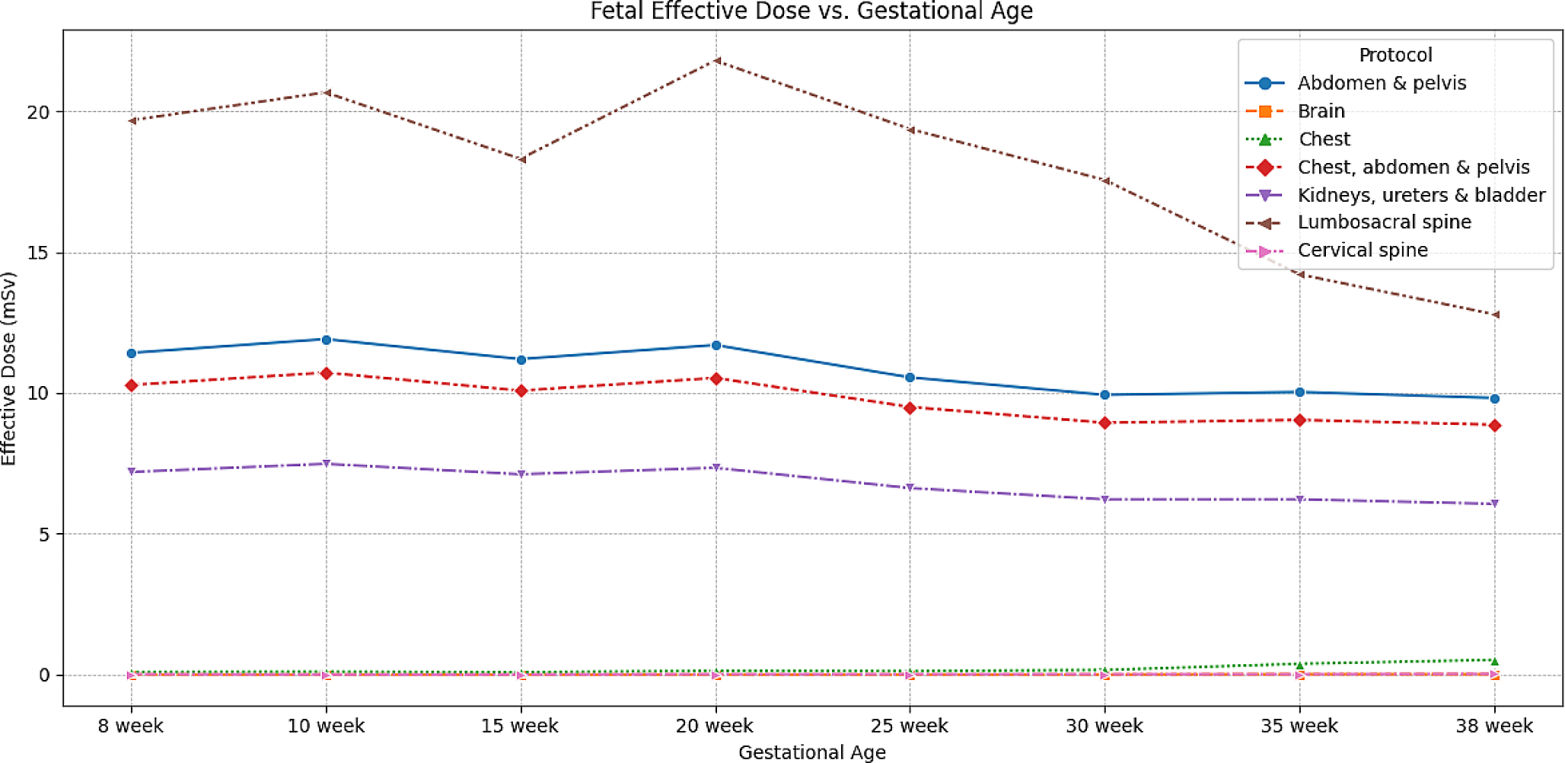
The estimated fetal effective dose for the varying gestational ages and protocols

As illustrated in Fig. 2, the calculated effective dose varies by gestational age and protocol. Of the protocols where the fetus is subjected to the primary beam, the lumbar spine protocol results in the highest fetal dose, ranging from 12.8 to 20.7 mSv. In contrast, the brain and cervical spine protocols expose the fetus only to scattered photons, yielding effective doses of less than 0.02 mSv. The chest protocol primarily exposes the fetus to scatter (with some minor primary beam exposure in the later gestational stages), with doses ranging between 0.1 and 0.5 mSv.

**Fig. 3:**
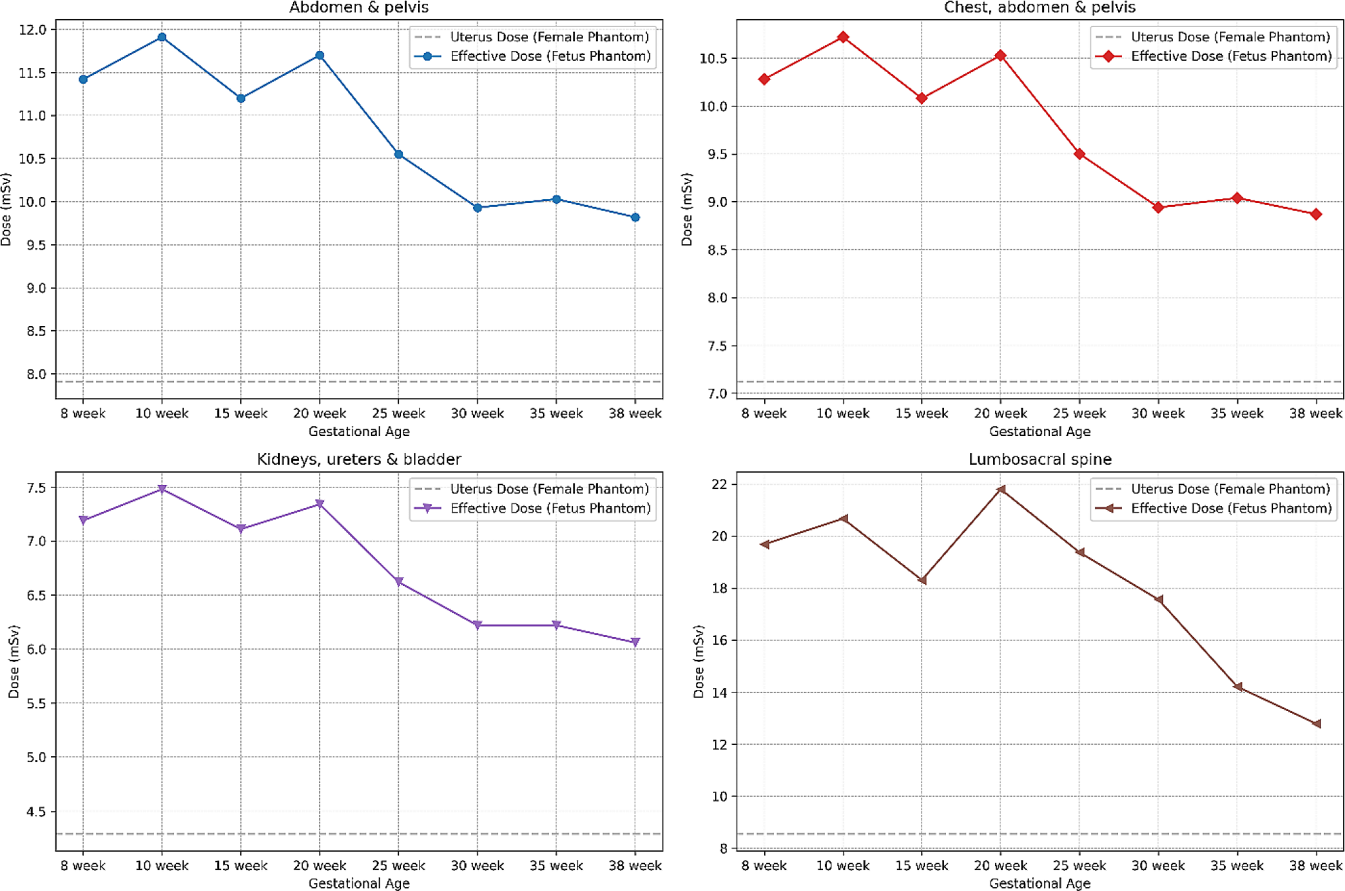
Comparison between female uterus dose and fetal dose for protocols where the fetus is subject to the primary beam

**Fig. 4:**
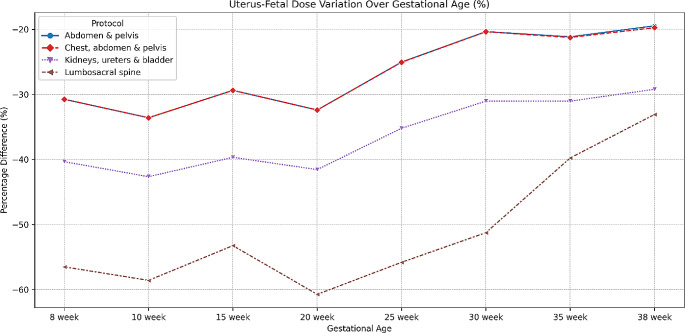
The percentage difference between using the female uterus and fetal doses

For protocols where the fetus was outside the primary beam, the disparity between the female uterus dose and the fetal dose was negligible. However, for protocols where the fetus was exposed to the primary beam and the doses surpassed 1 mSv, a comparison between the female uterus and fetal doses was conducted. Figure 3 illustrates the absolute difference between these doses. In all cases, using the uterus for dose estimation resulted in consistent underestimations of the effective dose, particularly notable in the lumbar spine protocol, as depicted in Fig. 4.

**Fig. 5:**
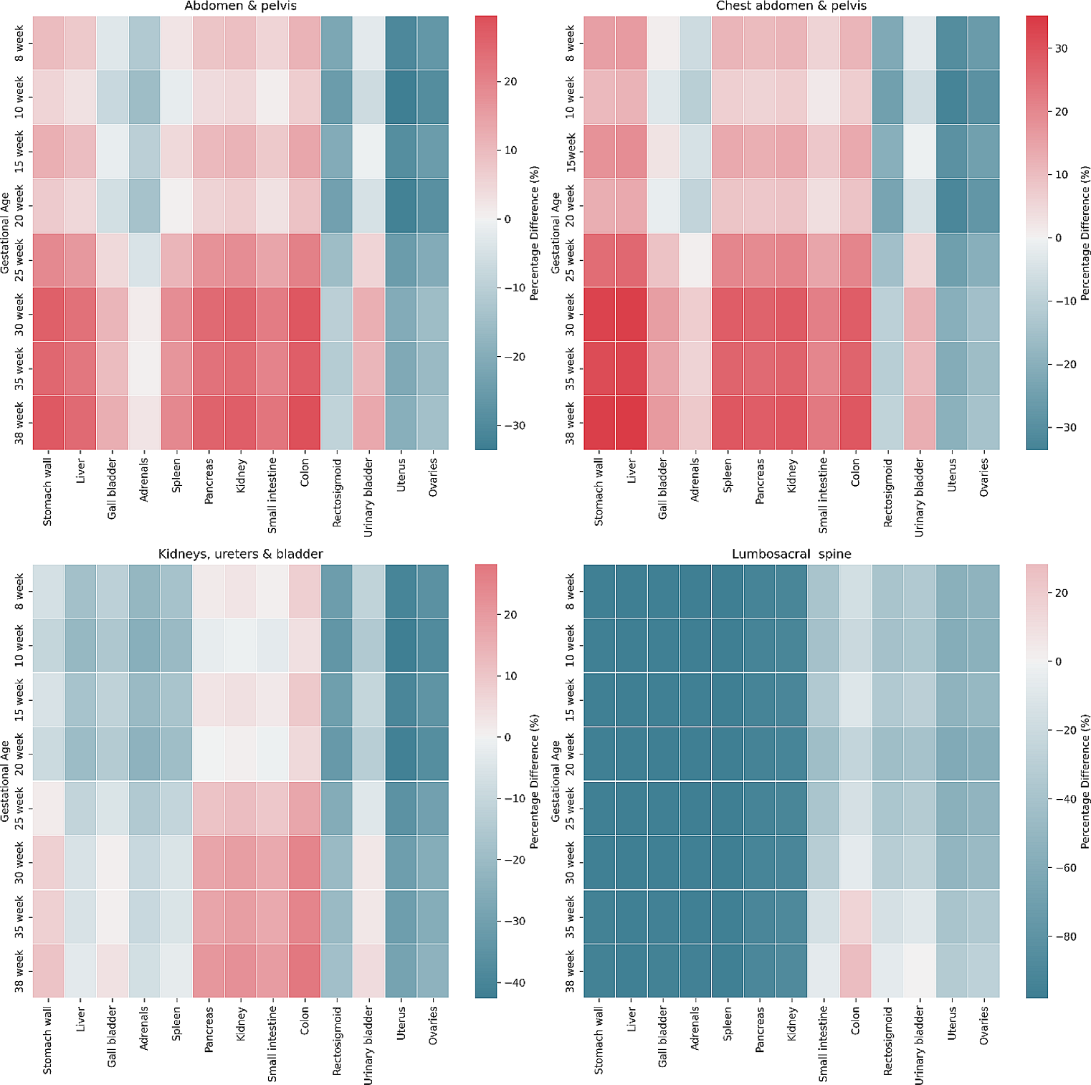
The percentage differences between organ doses in the female phantom and the effective dose for the fetus throughout the gestational period

Figure 5 compares the dose differences between the female abdominal/pelvic organs and the fetus across various gestational stages and protocols. Protocols where the fetus remains outside the primary beam, or where the dose difference between the uterus and the fetus is less than 1 mGy, have been omitted from the analysis. As noted earlier, the dose discrepancy between the female uterus and fetal effective dose is negligible in these scenarios. However, when the fetus is directly exposed to the primary beam, the organs that provide the closest estimate to the fetal dose for each gestational age and protocol are listed in Table 2.

**Table 2:** The organs with the lowest percentage difference for each protocol and gestational period

Gestational age (weeks)	Abdomen & pelvis	% Diff	Chest, abdomen & pelvis	% Diff	Kidney, ureter & bladder	% Diff	Lumbosacral spine	% Diff
8	Urinary bladder	−2.5	Gall bladder	0.8	Small intestine	1.0	Colon	−16.6
10	Small intestine	1.1	Small intestine	1.7	Kidney	−0.9	Colon	−20.6
15	Urinary bladder	−0.6	Urinary bladder	−0.7	Small intestine	2.1	Colon	−10.4
20	Spleen	0.2	Gall bladder	−1.6	Pancreas	−0.1	Colon	−24.7
25	Gall bladder	4.5	Adrenals	0.7	Stomach wall	1.4	Colon	−15.3
30	Adrenals	1.4	Adrenals	7.0	Gall bladder	1.0	Colon	−6.5
35	Adrenals	0.4	Adrenals	5.9	Gall bladder	1.0	Urinary bladder	−9.6
38	Adrenals	2.5	Adrenals	7.9	Spleen	−2.5	Urinary bladder	0.4

## Discussion

The protocols demonstrating the greatest variance are primarily those subjected to the beam exposure, notably the lumbosacral spine, followed by the chest, abdomen and pelvis, and kidney, ureters and bladder. Amongst these protocols the most significant variance is shown in the early gestational period, with a subsequent reduction in the difference illustrated from the 20th week. The significant variation in the radiation doses observed amongst these protocols can be attributed to the dynamic changes in fetal growth and positioning throughout the gestational period. As the fetus progresses in growth and development, its spatial alignment shifts concerning the maternal anatomy, which holds particular importance for these protocols near the expanding female abdomen.

In contrast, protocols including the brain and neck demonstrated the lowest radiation doses, Fig. 2. This can be attributed to the protocols’ significant distance from the reproductive organs and the developing fetus, consequently precluding them from being subjected to radiation beam exposure. The considerable distance between these specific regions and the maternal abdomen and attenuation by intermediary organs is a protective barrier; therefore, increased fetal size does not pose a potential risk factor for increased radiation exposure to these areas.

Where fetal phantoms are unavailable, using the uterus as a surrogate when the fetus is not exposed to the primary radiation beam is a reasonable approach. In these instances, the fetus will likely receive less than 1 mSv, and the associated risks are negligible. For the most part, personalised dosimetry is not warranted in these scenarios. However, for imaging protocols where the fetus is positioned within the primary beam’s path, Table 2 provides guidance into which organs serve as the most accurate surrogates for fetal dosimetry.

Across all protocols and gestational stages, the uterus consistently underestimated the fetal dose, often depicted as the least accurate surrogate organ. Depending on the protocol and gestational age, uterus dose estimation can be misleading, with underestimations between 20 and 60%. The lumbar spine protocol exhibits this most significantly, underestimating by 13.4 mSv. This may lead to an unintentional misclassification of risk associated with the procedure, especially in cases of multiple imaging events. Such misjudgment may result in alterations in patient care and potentially misinformed decisions.

Lopez-Rendon et al. observed a variation of −15.9 to 40.0% in the abdomen and pelvis protocol when using CT-Expo [11], a software that utilises computational methods and simple mathematical phantoms [8]. In contrast, this study observed a difference between −30.7% and −19.5%. The variation between the two studies might be due to the anatomical differences in the CT-Expo, voxelised phantoms, and NCICT phantoms and differences in the modelling of scanner output values.

Future research could build upon this study by comparing its findings with other commercially available software and patient-specific Monte Carlo simulations, such as those by Xie et al. and Lopez-Rendon et al. [11, 13]. The growing field of personalised organ dosimetry harnessing deep neural networks [14, 15], holds significant potential. Such advancements could improve the accuracy and efficiency of patient-specific dosimetry, eliminating the dependence on average patient phantoms used by most currently easy-to-use tools.

This study has several limitations. Firstly, the authors employed generic CTDI values representative of standard scans rather than those tailored for pregnant patients. While this approach impacts the typical fetal dose, the scaling of CTDI means the relative differences should be consistent. Secondly, the study didn’t account for Tube Current Modulation (TCM) when adjusting the mA across different patient regions, potentially influencing the dose disparity between the fetus and nearby organs. While there are no direct studies examining the uncertainties of NCICT. Lee et al. have outlined some of the limitations. Firstly, given the diverse sources available, the organ dose output is inevitably subjected to uncertainties as the application will not cover every scanner type, model, and subsequent update. Secondly, the ICRP phantoms cannot represent every patient accurately, and thus discrepancies will arise [12]. Additional patient factors, such as a full bladder, can alter the fetus’s position, introducing discrepancies in actual versus estimated doses [11]. Lastly, Lee et al. and Thierry-Chef et al. have further investigated the shared and unshared uncertainties regarding collecting retrospective data of CT parameters for the organ dose estimation [16, 17].

## Conclusion

Fetal dosimetry in CT imaging is especially important given the heightened radiosensitivity of the fetus. This research evaluated the practice of using the female uterus as a surrogate indicator for a fetal effective dose. The findings indicate significant shortcomings with this approach, especially when the fetus is positioned within the path of the primary radiation beam. These discrepancies, especially when leading to underestimations, might affect clinical decision-making and alter risk communications, impacting the understanding of associated risks. This study highlights the need for more accurate and detailed dosimetry methods for pregnant patients. Given the current landscape of software accessibility and ease of use, these findings suggest alternative organs that can serve as more representative surrogates for the fetus during different gestational stages.
